# Case report: Evaluation of cutaneous squamous cell carcinoma metastasized to lymph nodes using ^18^F-fluoro-2-deoxy-D-glucose positron emission tomography/computed tomography in a dog

**DOI:** 10.3389/fvets.2024.1429094

**Published:** 2024-07-26

**Authors:** Jin Seok, Sungin Lee

**Affiliations:** Department of Veterinary Surgery, College of Veterinary Medicine, Chungbuk National University, Cheongju, Republic of Korea

**Keywords:** cutaneous squamous cell carcinoma, flank, lymph node, positron emission tomography, 18F-fluorodeoxyglucose

## Abstract

**Introduction:**

18F-fluorodeoxy-2-deoxy-D-glucose (FDG) positron emission tomography (PET) is used with high sensitivity in human medicine for initial staging and treatment planning of cutaneous squamous cell carcinoma (SCC). To the best of our knowledge, 18F-FDG PET/computed tomography (CT) has not been used for canine cutaneous SCC with lymph node metastasis.

**Case presentation:**

A 13 year-old spayed female Maltese had rapidly growing flank SCC, which had previously recurred twice. Radiography revealed no metastases. On PET/CT imaging, increased FDG uptake was observed not only in the flank but also in the left axillary lymph node and left inguinal lymph node (standardized uptake value max [SUVmax]: 8.602, 5.354, and 1.96, respectively). Despite the evidence of metastasis, palliative skin mass resection with a 3-cm margin and lymph node dissection were performed. Histopathological examination confirmed the presence of metastases in both lymph nodes.

**Discussion:**

18F-FDG PET/CT is valuable for the detection of metastatic tumors in various organs. Cutaneous SCC can accumulate 18F-FDG, making it detectable on PET/CT. In this dog with flank SCC, 18F-FDG-PET/CT showed high SUVmax values, indicating its potential for tumor assessment. In veterinary medicine, SUVmax values of 2.5–3.5 are commonly used to identify metastatic lymph nodes in other cancers. Therefore, the interpretation of an SUVmax of 1.96 in an inguinal lymph node for metastatic involvement may be uncertain. Owing to the partial volume effect, 18F-FDG PET/CT has limited sensitivity in identifying LN metastases, particularly in cases of small lesions. Lower SUVmax values adjusted for smaller sizes may better distinguish between benign and malignant lymph nodes. Hence, combining differentiated SUVmax cut-offs based on lymph node size with CT assessment could enhance lymph node evaluation and assist in surgical planning.

## 1 Introduction

Squamous cell carcinoma (SCC) is a malignant neoplasm that originates from keratinocytes and accounts for 5% of canine skin tumors. Common predisposing factors include exposure to solar ultraviolet radiation. It predominantly affects older dogs at approximately 8 years of age. Common sites for SCC include the nail bed, scrotum, nasal planum, legs, and anus. Reports have also documented the occurrence of unpigmented flanks or abdomens in breeds such as Dalmatians and Beagles (1). The metastatic rate of cutaneous SCC in dogs remains unclear but is estimated to be approximately 5% (2). Tumors on the flank or abdomen are usually locally invasive, with low metastatic potential in the lungs or regional lymph nodes (LNs) (1).

Positron emission tomography (PET)/computed tomography (CT) is extensively used in human medicine for the diagnosis and staging of malignant tumors as well as the assessment of metastasis. This imaging technique combines CT for anatomical localization and PET for the imaging of biochemical metabolic changes. Typically, the radiopharmaceutical 18F-fluorodeoxy-2-deoxy-D-glucose (FDG) accumulates in areas of active glucose metabolism. This allows for the early detection of cancers, even those smaller than 1 cm, before visible changes occur based on biochemical abnormalities. Thus, 18F-FDG-PET/CT has a potential role in the early diagnosis and treatment planning of cutaneous SCC with high sensitivity in humans (3).

In veterinary medicine, PET/CT is used to image various tumors and provide valuable diagnostic information (2, 4, 5); however, to our knowledge, there have been no previous reports of cutaneous SCC in dogs. In one study, SCC was diagnosed in two of 14 dogs with standardized uptake value max (SUVmax) values of 12.8 and 17.1, respectively, and the location of the SCC was not mentioned (6). The novelty of this case report that it demonstrates the use of 18F-FDG PET/CT in a dog with histopathologically confirmed cutaneous SCC and regional LN metastases.

## 2 Case description

A 13-year-old Maltese patient visited our clinic for the evaluation of flank tumor metastases. The tumor had grown rapidly over the past 2 years. The mass initially appeared as a papilloma and was small. On February 28, 2023, the mass was surgically resected under local anesthesia at a local veterinary clinic. However, another mass subsequently recurred caudally to the surgical site and was resected using the same technique on September 22, 2023. After that, the masses recurred at both sites. No pathological diagnoses were made during either surgery. Subsequently, biopsies of both sites were performed at another local veterinary hospital, and SCC was diagnosed by histopathological examination (GreenVet, Yongin, Republic of Korea). The patient presented to our hospital with a bandage placed on the ruptured flank masses.

During physical examination, a ruptured and inflamed masses were found in the central part of the left flank, measuring 3.1 × 1.8 cm cranially and 4.5 × 2.6 cm caudally. The left axillary and inguinal LNs were palpably enlarged, particularly the left axillary node, which was approximately four times larger and significantly firmer than the left inguinal LN, measuring approximately 1 cm in diameter. Radiographs showed gallstones and nephroliths; however, there was no significant metastatic evidence related to the flank mass. Therefore, 18F-FDG PET/CT (Discovery-72 STE; General Electric Medical Systems, Waukesha, WI, USA) was planned for further metastatic evaluation. Before that, the dog underwent pre-anesthetic blood tests, including complete blood count, serum biochemical analysis, and venous blood gas analysis. Except for alkaline phosphatase (158 IU/L, reference range: 29–97 IU/L), reticulocyte count (150.8 × 10^3^/μl, reference range: 10.0–110.0 × 10^3^/μl), MCV (91.4, reference range: 61.6–73.5 fL), and MCHC (23.6, reference range: 32.0–37.9 g/dl), all other parameters were within normal ranges. FDG uptake may decrease when blood glucose levels exceed 200 mg/dl; however, in the present case, this patient was fasted for approximately 12 h and the glucose level was normal. Prior to the PET/CT scan, the dog was premedicated with midazolam (0.2 mg/kg; Midazolam, Bukwang Pharm. Co., Ltd., Seoul, South Korea), and a urinary catheter was placed. Anesthesia was induced using propofol and maintained with isoflurane. Before the PET scan, CT images (pre- and post-contrast) were obtained using a multidetector CT scanner with settings of 100 mAs and 120 kVp, and a slice thickness of 1.25 mm. Contrast-enhanced scans were performed after intravenous administration of iohexol at a dose of 880 mg/kg. The interval between the 18F-FDG injection and the start of scanning was 45 min, during which the patient was under anesthesia.

**Figure 1:**
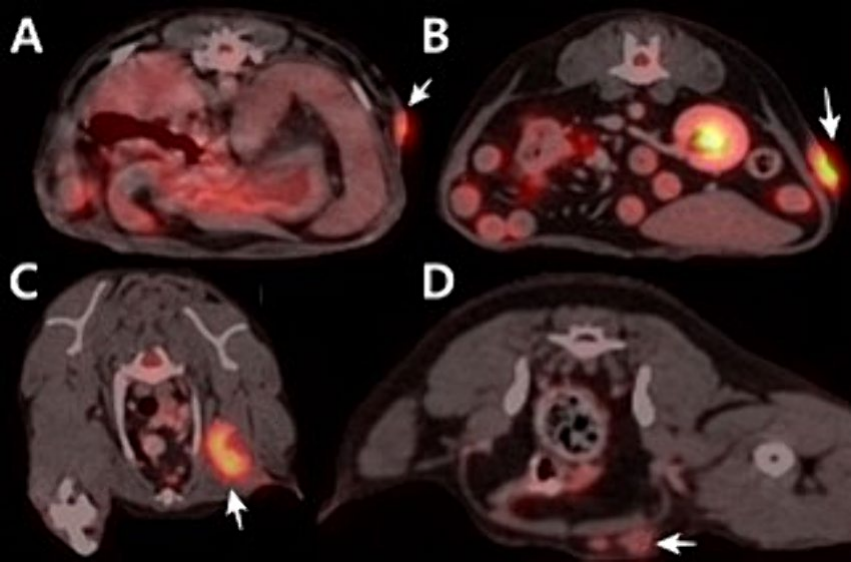
Positron emission tomography/computed tomography using 18F-fluorodeoxyglucose fusion images of the dog. **(A)** Cranial part of flank mass. SUVmax is 5.354. **(B)** Caudal part of flank mass. SUVmax is 8.062. **(C)** Axillary lymph node. SUVmax is 7.453. **(D)** Inguinal lymph node. SUVmax is 1.961.

**Figure 2:**
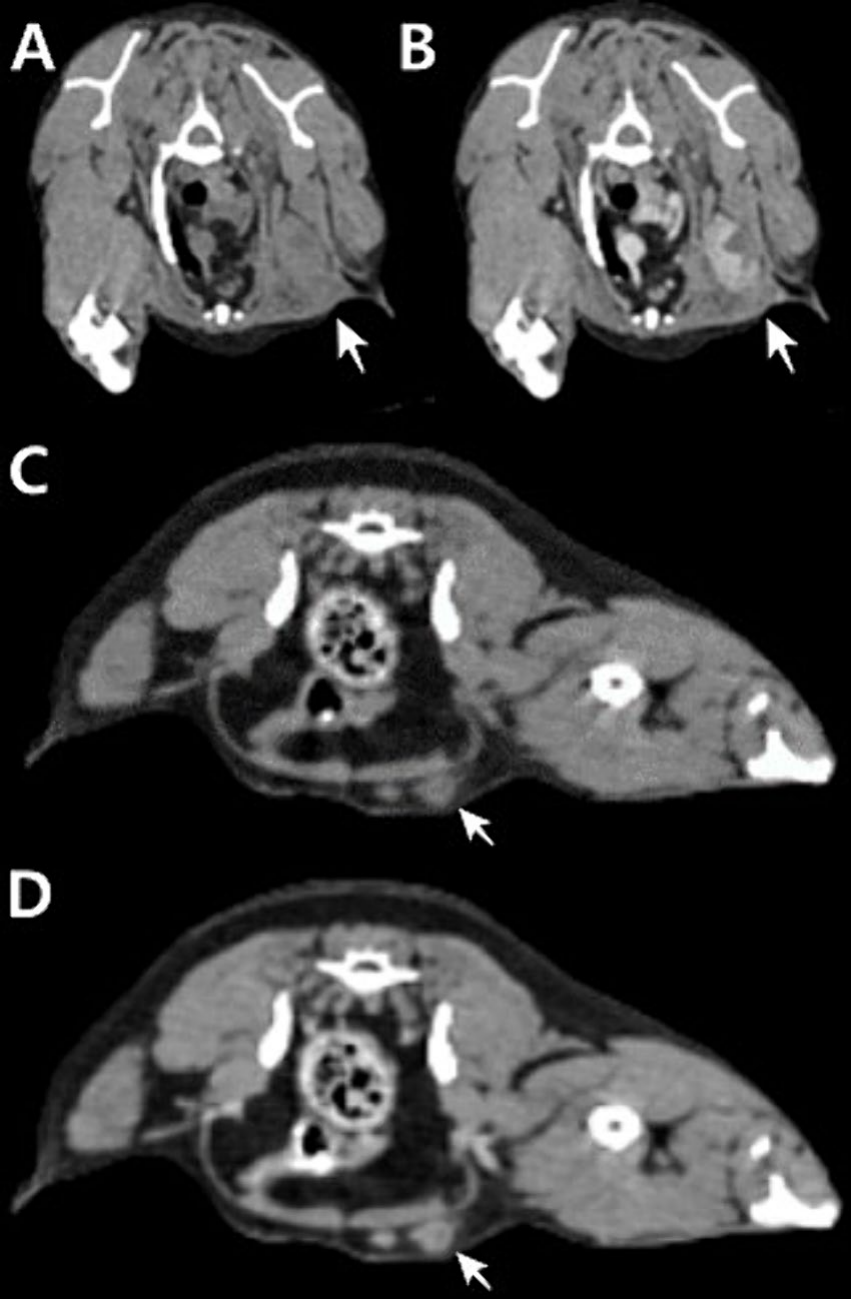
Transverse planar computed tomography (CT) images of the left axillary lymph node measuring 3.74 × 1.58 × 2.5 cm **(A,B)** and left inguinal lymph node measuring 7.49 × 8.67 × 6.08 mm **(C,D)**. Pre-contrast CT image **(A,C)**. **(B)** Post-contrast CT image showing heterogeneous enhancement with multifocal areas of necrosis and apparent invasion into the surrounding tissue. **(D)** Post-contrast CT image showing subtle contrast enhancement.

In 18F-FDG PET/CT scans, the region of interest was manually defined and the standardized uptake value (SUV) was calculated using the following formula: Average tissue activity concentration (MBq/mL) × body weight (g)/injected dose (MBq). This numerical value provides a semiquantitative assessment of 18F-FDG uptake and serves as an indicator of metabolic activity within the respective regions. The PET/CT scan showed increased 18F-FDG uptake in the left flank mass, the left axillary LN, and left inguinal LN. The corresponding SUVmax values were 5.354 (cranial part of the flank masses), 8.602 (caudal part of the flank masses), 7.453, and 1.961 (Figure 1). CT revealed that the depth of the left flank masses was approximately 6.56 mm. Post-contrast CT imaging showed heterogeneous enhancement in the left axillary LN, measuring 3.74 × 1.58 × 2.5 cm, with apparent invasion into the surrounding tissues (Figures 2A,B). The left inguinal LN measured 7.49 × 8.67 × 6.08 mm with subtle contrast enhancement on post-contrast CT imaging (Figures 2C,D). In addition, the right axillary lymph node (LN) measured 7.65 × 3.22 × 4.63 mm, with homogeneous contrast enhancement on post-contrast CT images and an SUV max of 0.6737 on PET/CT. The right inguinal LN measured 4.49 × 5.18 × 2.59 mm, also showing homogeneous enhancement on post-contrast CT, with an SUV max of 1.526.

**Figure 3:**
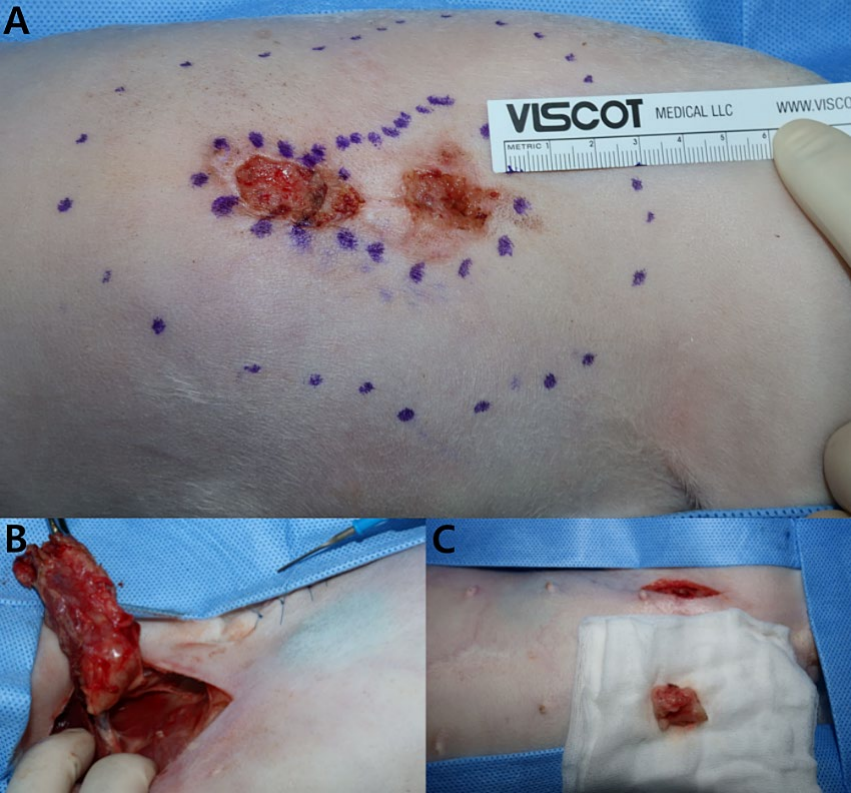
Flank skin mass resection and lymphadenectomy. **(A)** The flank mass was excised with a 3-cm margin, including the subcutaneous fat. **(B)** The left axillary lymph node infiltrating the surrounding tissues was excised, noting the mass exceeding 3 cm in size and its palpable firmness. **(C)** The left inguinal lymph node was resected together with the adjacent tissue.

Despite the detection of LN metastasis on PET/CT, the patient underwent skin mass resection and a two-site lymphadenectomy for palliative purposes (Figure 3). Based on the previous histopathological examination revealing SCC and considering the high SUVmax (8.602) and history of two recurrences, the masses were deemed to be highly malignant. Therefore, the excision of the flank masses including the subcutaneous fat was planned with 3-cm margin. After premedication with midazolam (0.2 mg/kg; Midazolam, Bukwang Pharm. Co., Ltd., Seoul, South Korea), propofol (6 mg/kg; Provive, Myungmoon Pharm. Co., Ltd., Seoul, South Korea) was administered intravenously to induce anesthesia, which was maintained with isoflurane (Terrell, Piramal Critical Care, Bethlehem, PA, USA). Local infiltration anesthesia was applied on the left flank with a mixture of bupivacaine and lidocaine. Intraoperative pain control was achieved with a continuous infusion of remifentanil (5 μg/kg/min; Tivare BCWORLD Pharm. Co., Ltd., Yeojoo, South Korea).

**Figure 4:**
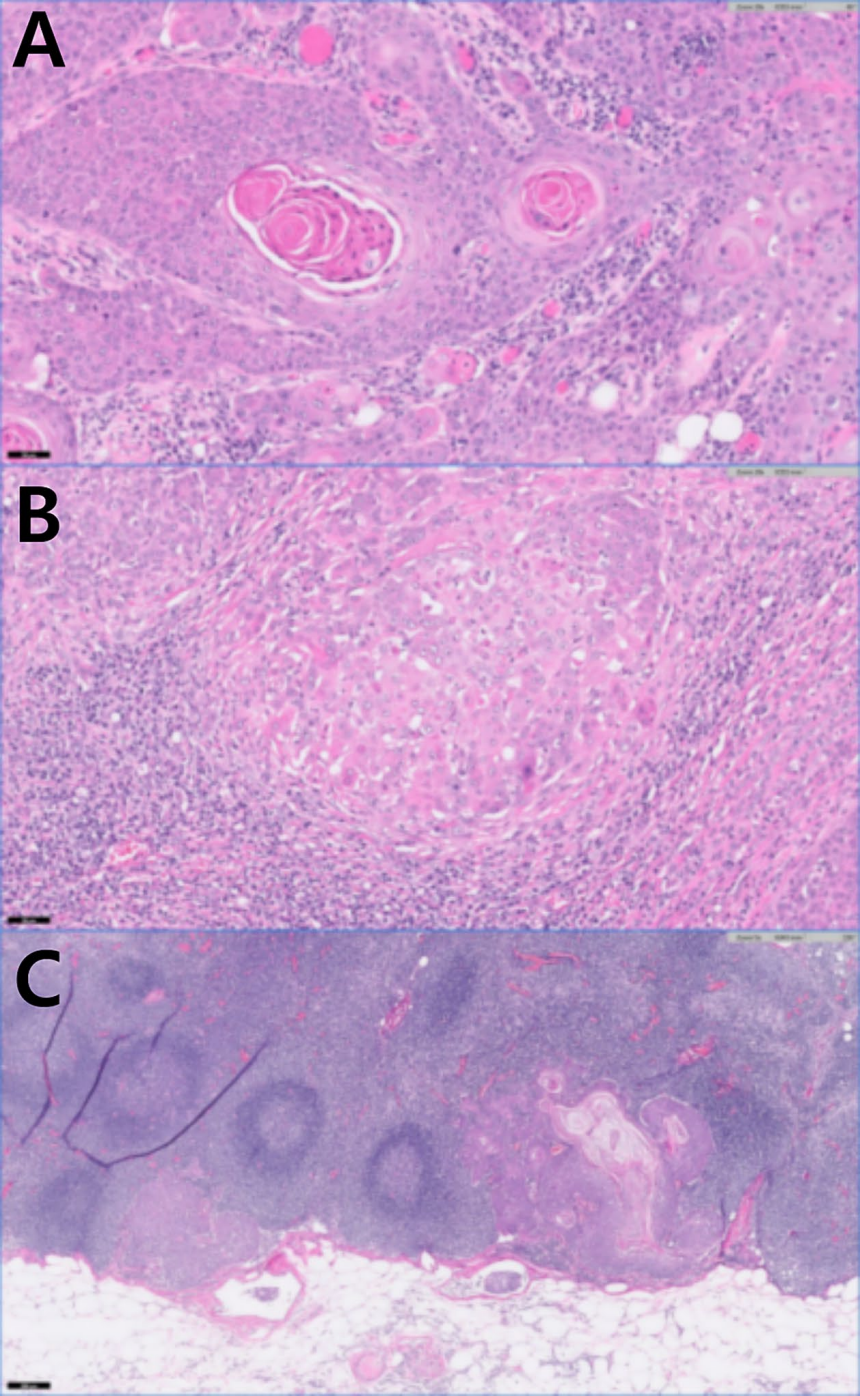
Histopathology of the left flank mass after skin mass resection and lymphadenectomy. **(A)** Keratin pearls present in squamous cell carcinoma (H&E stain, original magnification×20, scale bar 100 um). **(B)** Left axillary lymph node with squamous cell carcinoma (H&E stain, original magnification×20, scale bar 50 um). **(C)** Left inguinal lymph node with squamous cell carcinoma (H&E stain, original magnification5, scale bar 200 um).

Histopathological examination of the left flank tumors confirmed SCC extending from the dermis to the subcutis (IDEXX Laboratories, Inc., USA). Keratin pearls were observed within numerous lobules, trabeculae, and islands (Figure 4A). Anisocytosis and anisokaryosis were moderate, with mitotic counts greater than 30. The surgical margins were complete; however, extensive carcinomatous spread to both the left axillary and inguinal LNs was observed (Figures 4B,C). Moderate anisocytosis and anisokaryosis were noted, with approximately 25 mitoses per 10 high-power fields in the inguinal LN and more than 30 mitoses in the axillary LN. There were frequent areas of necrosis throughout the neoplastic tissue. Neoplastic cells were present in the angiolymphatic vessels surrounding the LNs.

On the day after surgery, subcutaneous emphysema developed on the left flank, which resolved after 1 week of compression bandaging. Sutures were removed 14 days post-surgery; no signs of inflammation, tissue edema, or seroma were observed at any surgical sites. The owner declined further metastasis assessment and requested referral to nearby local animal hospital for subsequent management. About 2 months after the surgery, the X-ray showed nodules suspected to be lung metastases, and we were informed that chemotherapy would be started at the local hospital. However, we were not given any information about the specific chemotherapy protocol.

## 3 Discussion

Cutaneous SCC of the flank typically grows slowly and is locally invasive, with a low metastasis rate. In this case, however, the tumors had rapidly progressed over the past year. The tumors were resected thrice with incomplete margins. PET/CT revealed metastases in the left axillary and inguinal LNs, confirmed by histopathological examination. In humans, failure to completely excise cutaneous SCC increases the likelihood of local recurrence and metastasis (7). Initially, because of their papilloma-like appearance and small size, the tumors were resected with incomplete margins, and histopathological examination was omitted under the assumption that they were papilloma-like lesion. This decision was believed to have contributed to their rapid growth and metastasis. In human medicine, surgical margins for the excision of SCC are determined based on the tumor size, histological grade, and location. However, in veterinary medicine, there are no evidence-based criteria for determining the extent of surgical margins for cutaneous SCC excision, except in the nasal planum (8). In the absence of specific margin recommendations for other sites, T3 and T4 (World Health Organization’s tumor-node-metastasis staging system) cases are recommended to have a minimum margin of 2 cm, which is similar to that of nasal planum SCC (9). In this case, the tumors depth exceeded 6 mm and the cranial and caudal parts were >2 cm in diameter. In addition, there was a history of recurrence and LN metastases, classifying this case as high-risk cutaneous SCC based on the human medicine criteria (10). Because obtaining a clear surgical margin is important for a good prognosis, we resected the flank masses with a wide margin of 3 cm and excised the LNs with suspected metastases (Figure 3).

18F-FDG PET/CT is a non-invasive technique that is commonly used for detecting tumor metastasis. The principle behind its use is the Warburg effect, wherein cancer cells have increased glucose utilization, resulting in elevated FDG uptake through the upregulated expression of GLUT-1 and hexokinase (11). Cutaneous SCC cells express GLUT-1 and can accumulate 18F-FDG (3, 12). In humans, PET/CT is predominantly used to diagnose SCC in areas such as the head, neck, oropharynx, and esophagus, with occasional use for lesions in the trunk and feet (3, 13). In a study of 23 patients diagnosed with cutaneous SCC, all had FDG-positive scans and the mean SUVmax was approximately 10.2 (3). In dogs, carcinomas have higher SUV values compared to sarcomas (SUVmax, 2.0–10.6), with reported SUVmax ranging from 7.6 to 27.0 (6). In one study, 2 of 14 dogs were identified as having SCC with SUVmax values of 12.8 and 17.1, respectively; however, their specific locations were not mentioned (6). Flank SCC in dogs, as observed in this case, had a significantly high SUVmax, indicating the potential for the evaluation of these tumors using 18F-FDG-PET/CT.

LN assessment is critical for staging and treatment planning. In dogs, the normal range of SUVmax for LNs is approximately 0.5–2.0 (14). There is no precedent for assessing LN metastasis in canine cutaneous SCC using PET/CT. Typically, an SUVmax between 2.5 and 3.5 is used as the cutoff value for identifying metastatic LNs (11, 15–17). In humans, metastatic LNs in cutaneous SCC typically exhibit SUVmax values greater than 7, distinguishing them from false-positive conditions, such as lymphadenitis. Conversely, true-negative cases have SUVmax values below 1.4 (18). Therefore, an SUVmax of 1.96 in a LN may be considered ambiguous for diagnosing metastatic involvement. The limited sensitivity of 18F-FDG in identifying LN metastasis is because of the partial volume effect, where radioactivity from small lesions under 10 mm spills into the background, leading to the underestimation of FDG uptake (19). This effect has also been reported in canine mammary gland tumors (20). Several studies have suggested the use of differential SUVmax cut-offs based on LN size, typically using thresholds of 7 or 10 mm, to enhance the sensitivity and accuracy of PET/CT in detecting metastatic LNs (19, 21, 22) and this has also been mentioned in veterinary medicine (23). In this patient, the left inguinal LN size on CT was 7.49 × 8.67 × 6.08 mm, with the longest axis being less than 10 mm. To differentiate between benign and malignant LNs, it may be more appropriate to apply a lower SUVmax that is tailored to smaller LNs. CT is commonly used to show enlarged lymph nodes as an indication of metastasis. However, it is generally accepted that normal-sized lymph nodes can also contain metastases, while enlarged lymph nodes may sometimes be free of metastasis. The evaluation of CT scans with differentiated SUVmax cut-off values based on LN size may provide a more accurate assessment of LN status and assist in selecting biopsy sites during surgery.

In conclusion, this case demonstrates, for the first time, that flank cutaneous SCC metastasizes to regional LNs in dogs with high FDG uptake on PET/CT. Therefore, diagnosing cutaneous SCC and assessing metastasis in dogs is a feasibility of PET/CT, similar to its application in humans. In veterinary medicine, although there are no defined SUVmax cutoff values for malignant LNs, it is important to consider that LNs smaller than 10 mm may have an FDG uptake lower than the SUVmax of 2.5, even when histopathologically confirmed as malignant metastatic LNs. Although further studies are needed, the interpretation of a lower SUVmax value as the cut-off for LNs <10 mm could enhance the sensitivity of the detection of LN metastasis.

## Data availability statement

The original contributions presented in the study are included in the article/supplementary material, further inquiries can be directed to the corresponding author.

## Ethics statement

Ethical approval was not required for the studies involving animals in accordance with the local legislation and institutional requirements because this is the case report of a clinical patient, not an experimental research. Written informed consent was obtained from the owners for the participation of their animals in this study.

## Author contributions

JS: Writing – review & editing, Writing – original draft, Data curation, Conceptualization. SL: Writing – review & editing, Writing – original draft, Supervision, Funding acquisition, Data curation, Conceptualization.
